# Impact of Right-Sided-Catheter-Based Valve Implantation on Decision-Making in Congenital Heart Disease

**DOI:** 10.1007/s11886-016-0712-2

**Published:** 2016-02-25

**Authors:** Joanna Ghobrial, Jamil Aboulhosn

**Affiliations:** Ahmanson/UCLA Adult Congenital Heart Disease Center, 200 Medical Plaza Driveway #365, Los Angeles, CA 90024 USA

**Keywords:** Congenital heart disease, Tricuspid regurgitation, Tricuspid stenosis, Pulmonary regurgitation, Pulmonary stenosis, Melody valve, Sapien valve, Transcatheter valve replacement, Tetralogy of Fallot

## Abstract

There is a growing appreciation for the adverse long-term impact of right-sided valvular dysfunction in patients with congenital heart disease. Although right-sided valvular stenosis and/or regurgitation is often better tolerated than left-sided valvular dysfunction in the short and intermediate term, the long-term consequences are numerous and include, but are not limited to, arrhythmias, heart failure, and multi-organ dysfunction. Surgical right-sided valve interventions have been performed for many decades, but the comorbidities associated with multiple surgeries are a concern. Transcatheter right-sided valve replacement is safe and effective and is being performed at an increasing number of centers around the world. It offers an alternative to traditional surgical techniques and may potentially alter the decision making process whereby valvular replacement is performed prior to the development of long-term sequelae of right-sided valvular dysfunction.

## Introduction

The progress of congenital heart disease (CHD) surgical interventions over the past few decades has allowed more children to survive well into adulthood. Most of these patients will require multiple surgical procedures over their lifetime. This can be associated with increased morbidity and mortality [1, 2] due to chest adhesions, bleeding, cardiac ischemia, arrhythmia burden, heart failure, and multi-organ dysfunction [3–7]. Since the introduction of the first balloon expandable valve in the pulmonary position by Bonhoeffer et al [8] in 2000, advances in interventional cardiology and percutaneous valve replacement have revolutionized the management of these patients. The availability of these minimally invasive and effective therapies may allow for earlier treatment of right-sided valvular disease before the onset of irreversible ventricular remodeling and dysfunction. Moreover, transcatheter options can reduce the need for multiple surgical interventions over a patient’s lifetime, therefore affecting the morbidity of this growing patient population [9]. We herein discuss the impact of existing and future catheter-based valve implantation techniques for dysfunctional right-sided valves.

## Pathophysiology and Clinical Presentation of Right-sided Valvular Dysfunction in Congenital Heart Disease

### Pulmonary Valve

Pulmonary valve disease in the congenital population can be native or post-operative. Native pulmonary valve dysfunction may be isolated, as in congenital pulmonary stenosis, or may be a part of a recognized condition such as tetralogy of Fallot. Surgical valve or conduit placement is often not performed in infancy or early childhood in preference to procedures that alleviate pulmonic stenosis such as pulmonary valvotomy and subannular or transannular patch placement. These techniques invariably leave the patient with chronic severe pulmonary regurgitation. Surgical pulmonary valve interventions usually consist of valve replacement with either bioprosthetic valves or valved conduits. Rarely, patients may undergo mechanical pulmonic valve replacement. Bioprosthetic valves and conduits (usually homografts) invariably develop progressive dysfunction within 10–20 years of implantation with resultant stenosis and/or regurgitation [10–12].

Although pulmonary valve dysfunction is generally well tolerated, patients often develop progressive exertional limitations and may develop ventricular and supraventricular arrhythmias. Decompensated congestive heart failure is a late manifestation that usually occurs well after the development of exertional symptoms. In patients with progressive stenosis, right ventricular hypertrophy develops as a compensatory mechanism for the increased pressure with resultant right ventricular diastolic dysfunction and eventually systolic dysfunction ensues [13]. Severe chronic pulmonary regurgitation results in right ventricular volume overload which leads to ventricular dilatation, progressive systolic and diastolic dysfunction, and tricuspid regurgitation due to annular dilatation [14]. Ventricular and atrial arrhythmias are likely to occur in patients with both stenosis and regurgitation and result in an increased risk of sudden death in this population [7, 15].

The timing of pulmonary valve replacement in these patients is controversial [16, 17]. Several studies have evaluated the timing of pulmonary valve replacement in patients with severe chronic pulmonary regurgitation and have suggested that normalization of right ventricular volume will not occur in patients with severely dilated right ventricles (right ventricular end-diastolic volume (RVEDV) >150 ml/m^2^ or right ventricular end-systolic volume (RVESV) >80 ml/m^2^) [18–23]. Therefore, it is generally recommended that patients undergo pulmonary valve replacement prior to severe right ventricular dilation or systolic dysfunction. However, there is little evidence that such an approach results in improved clinical outcomes, such as reduced arrhythmia burden or decreased mortality. Furthermore, it is not clear that different etiologies of pulmonic regurgitation behave in the same pathophysiological manner, and thus thresholds developed for regurgitation following repair of tetralogy of Fallot may not apply to those patients with initial isolated pulmonic stenosis [24]. It does appear likely that earlier PVR results in improved exercise tolerance, especially in patients with predominant pulmonary stenosis [17, 25].

### Tricuspid Valve

Native tricuspid valve dysfunction in patients with congenital heart disease is often associated with Ebstein anomaly, which may result in chronic severe tricuspid regurgitation. Severe tricuspid regurgitation is often well tolerated for decades. However, much like pulmonic regurgitation, eventual clinical sequelae will emerge. These include the development of ventricular and supraventricular arrhythmias, elevated central venous pressure with resultant multi-organ congestion (congestive hepatopathy, renal dysfunction, splenomegaly), and progressive right ventricular dysfunction. Native tricuspid valve regurgitation may occur as a result of progressive right ventricular and tricuspid annular dilation or in patients with severely elevated right ventricular systolic pressure due to severe pulmonary hypertension or right ventricular outflow obstruction. Therefore, it is common for patients with RVOT dysfunction, as in repaired tetralogy of Fallot or pulmonic stenosis, to develop secondary tricuspid regurgitation. Surgical techniques for tricuspid valve repair include the placement of annular bands or rings as well as more complex operations to relocate or augment the tricuspid valve leaflets (as in Ebstein’s). Progressive tricuspid regurgitation is common during long-term follow-up of these patients and manifests in a similar clinical manner to native tricuspid regurgitation. If surgical valve replacement is warranted, bioprosthetic valves are preferred, although there is limited data on the safety and efficacy of mechanical prostheses. Bioprosthetic tricuspid valves can become dysfunctional over time and generally require replacement within a decade. Progressive regurgitation and/or stenosis may occur with the clinical sequelae of elevated central venous pressure and reduced cardiac output [26, 27].

## Diagnostic Methods of Right-Sided Valvular Disease in Congenital Heart Disease

### Echocardiography

**Fig. 1:**
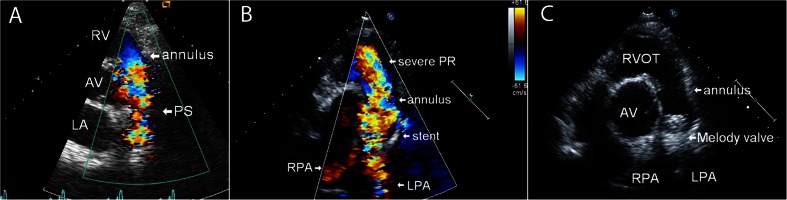
**a** Transthoracic echocardiographic image (parasternal short axis view) of the right ventricular outflow tract (RVOT) showing turbulent color Doppler flow across a stenotic pulmonary valve. AV = aortic valve. **b** Parasternal short axis view of RVOT with an indwelling stent in the main pulmonary artery well above the level of the pulmonary valve annulus and color Doppler flow showing severe pulmonary valve regurgitation (PR). Note the regurgitant reverse color Doppler flow in the left pulmonary artery (LPA) and right pulmonary artery (RPA). **c** Parasternal short axis view of the RVOT showing a Melody valve successfully deployed in the MPA

Echocardiography is a widely available and cost-effective tool for the assessment of congenital cardiac structure and function. It is universally utilized for the diagnosis of stenotic or regurgitant valvular lesions using qualitative measures such as color Doppler (Fig. 1), and density and contour of regurgitant signals, as well as quantitative measures such as vena contracta, effective regurgitant orifice area, and velocity of flow. The echocardiographic exam for right-sided heart lesions also includes assessment of right and left ventricular size and function, right ventricular outflow tract (RVOT) and tricuspid annular dimensions, pulmonary arterial pressure, right and left atrial filling pressures, and systemic and pulmonary blood flow [28–31]. The addition of 3D echo imaging facilitates the quantitative assessment of right ventricular volume and ejection fraction. The use of tissue Doppler and speckle tracking for assessment of systolic and diastolic function, including regional and global right ventricular strain, is an area of growing interest [32]. Echocardiography typically provides excellent visualization of the tricuspid valve but is less optimal for imaging the pulmonic valve, especially in adult patients. Moreover, echocardiography is widely utilized for the assessment of prosthetic valve function. The comprehensive use of a non-invasive and cost-effective tool such as echocardiography has obviated the need for diagnostic invasive catheterization in most patients with congenital heart disease.

### Computed Tomography Angiography

Cardiac tomography angiography (CTA) has become an integral part of the evaluation of patients with aortic valve disease prior to transcatheter aortic valve replacement (TAVR). CTA is not as widely utilized for the evaluation of patients with congenital heart disease, as many centers prefer to use cardiac MRI due to concerns over radiation and its limitations in functional assessment due to the lack of blood velocity measurements. Also, CTA requires the injection of iodinated contrast, which is nephrotoxic, and therefore the use of this modality is limited in patients with advanced renal dysfunction. However, ECG-gated chest CTA can provide excellent spatial and temporal resolution and is extremely useful in the assessment of right ventricular outflow tract abnormalities, both native and post-operative [33]. Moreover, CTA allows for visualization of the coronary arterial anatomy and its relationship to the right ventricular outflow tract and pulmonary artery. Therefore, CTA can be a useful tool for procedural planning in patients with congenital valvular heart disease [34]. The advent of 3D printing from cross-sectional imaging data allows for individualized planning of complex structural interventions [35].

### Cardiac Magnetic Resonance Imaging

Cardiac Magnetic Resonance Imaging (CMR) is widely used for the assessment of congenital heart disease, as it provides anatomic data as well as qualitative and quantitative measurements of valvular regurgitation and/or stenosis. In addition, it provides accurate ventricular dimensions and function. CMR is especially useful for measuring pulmonary regurgitant volume and right ventricular volumes, which are critical parameters for determining the optimal timing for intervening on pulmonary valve regurgitation [36–39]. CMR is non-invasive and does not expose patients to radiation; however, it is time consuming, not universally available, and more expensive than echocardiography and is contraindicated in some patients with pacemakers and defibrillators.

### Cardiac Catheterization and Invasive Angiography

Diagnostic cardiac catheterization was once utilized universally in patients with congenital heart disease but has been supplanted over the past 30 years by the aforementioned non-invasive imaging tools. In cases where the non-invasive data is inconclusive or contradictory, diagnostic catheterization may be necessary. In patients with right-sided congenital valvular disease, catheterization provides direct pressure measurements, facilitates angiography, and allows for accurate measurement of shunts. Moreover, in patients with pulmonary hypertension, catheterization allows for the calculation of pulmonary arterial resistance and determination of pulmonary vasoreactivity. Additionally, invasive hemodynamic assessment during steady state alterations, such as volume loading, stress testing, or infusion of inotropic medications can provide valuable information. That being said, the majority of patients today undergo invasive catheterization mainly for interventional purposes; however, the hemodynamic data gathered pre- and post-intervention is integral to their management.

## Catheter-Based Therapies

Indications for intervention are often based on symptomatology, severity of the stenosis or regurgitation, right ventricular volume and function, and the burden of arrhythmias [22, 38, 40].

### Pulmonary Valve

**Fig. 2:**
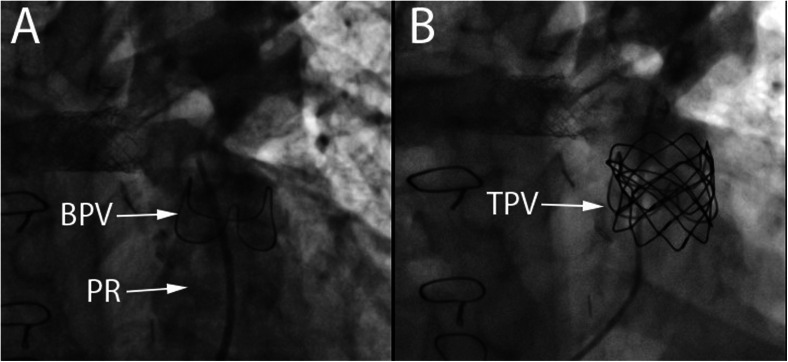
**a** Antero-posterior (AP) angiographic view of a bioprosthetic pulmonary valve (BPV) with contrast injection in the pulmonary artery showing severe pulmonary regurgitation and a stent in the right pulmonary artery. **b** AP view of the same bioprosthetic valve after transcatheter pulmonary valve placement (TPV) of a Melody valve. No residual pulmonary regurgitation is seen

**Fig. 3:**
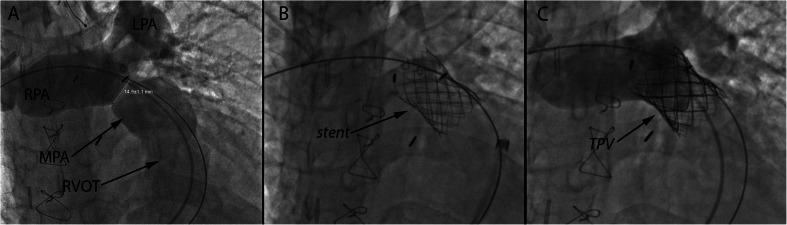
**a** Antero-posterior and cranial projection of a contrast injection into the main pulmonary artery (MPA) demonstrating severe pulmonary regurgitation into the native right ventricular outflow tract (RVOT). The right and left pulmonary arteries (RPA and LPA) are labeled, with a narrowing just proximal to the branching of the MPA measuring approximately 14.9 mm. Note sternal wires from previous median sternotomy. **b** Bare metal stent placed across the narrowed MPA. **c** Image following Melody transcatheter pulmonary valve (TPV) placement within the stent. Contrast injection in the MPA shows no residual pulmonary regurgitation

**Fig. 4:**
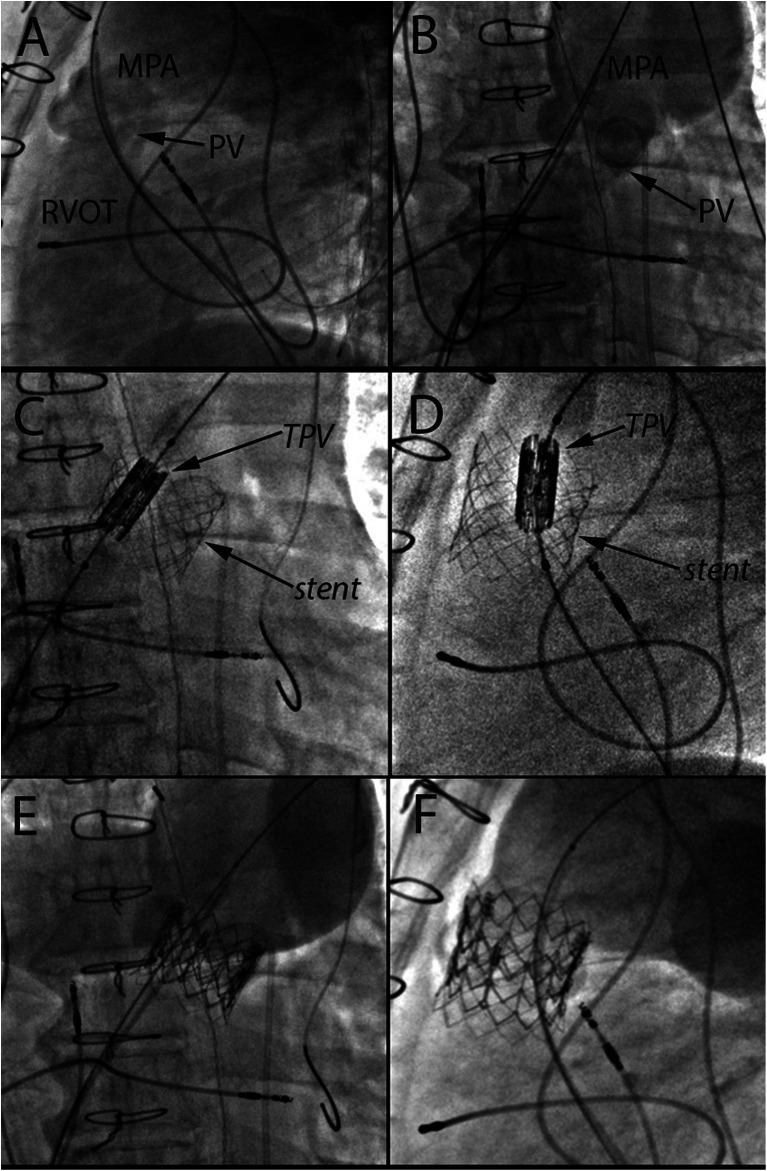
**a** Lateral angiographic view of a native right ventricular outflow tract (RVOT) with a wire placed in the distal left pulmonary artery and catheter in a dilated main pulmonary artery (MPA) showing severe regurgitation across a dysfunctional native pulmonary valve (PV). **b** Antero-posterior (AP) angiographic view of the same native RVOT showing severe PR. **c** AP fluoroscopic view of the Sapien XT transcatheter pulmonary valve (TPV) positioned within a stent across the native pulmonary valve. **d** Lateral view of the Sapien valve delivery system positioned across the pre-stented PV. **e** AP view of deployed Sapien valve within the stented PV. **f** Lateral view of the deployed Sapien valve within the pre-stented PV

Transcatheter pulmonary valve (TCPV) replacement can alleviate RVOT conduit dysfunction whether due to stenosis, regurgitation, or both, thereby delaying the need for open heart surgery and essentially decreasing the number of operations with their associated morbidities [41, 42]. The Melody (Medtronic) TCPV was the first valve to receive FDA approval, initially under humanitarian device exemption in 2010 and thereafter for clinical use in 2014. The Melody valve is widely used for the treatment of dysfunctional right ventricular to pulmonary artery (RV-PA) conduits and bioprosthetic valves and more recently has been used to treat dysfunctional native RVOT lesions (Figs. 2 and 3). Due to the size of the deployment system (22 Fr), when used via a transvenous approach, it is restricted to patients weighing more than 20–30 kg [41, 43]. This limitation does not apply when a hybrid surgical approach is utilized. Currently, the first generation Sapien (Edwards Lifesciences) TCPV is under investigation, also with a 22- and 24-F system (COMPASSION Clinical Trial NCT00676689). The later generations of the Sapien valve (Sapien XT and Sapien 3) are FDA-approved for use in the aortic position and are being used by multiple centers for off-label implantation in the pulmonary position (Fig. 4). The larger-diameter Sapien valves (26 and 29 mm) allow for implantation in large dysfunctional native RVOTs. The Sapien XT is also under clinical investigation currently for the pulmonary position (COMPASSION III Clinical Trial NCT02302131).

Short-term outcomes of the Melody valve have proven the durability of the valve [44–50]. Furthermore, there is significant hemodynamic and clinical improvements after Melody valve placement due to right ventricular remodeling, decrease in end diastolic volumes, increase in cardiac output, and subsequently an improvement in exercise tolerance [17, 25, 44, 48, 49, 51–54, 55•]. This effect is seen more significantly in patients with RVOT obstruction and/or regurgitation versus those with only regurgitant lesions [25]. Favorable longer-term outcomes were seen after a median follow-up of 4.5 years in 148 patients in the US melody valve investigational device exemption trial, with a 5-year freedom from re-intervention of 76 % and a 5-year freedom from explant of 92 % [56].

**Fig. 5:**
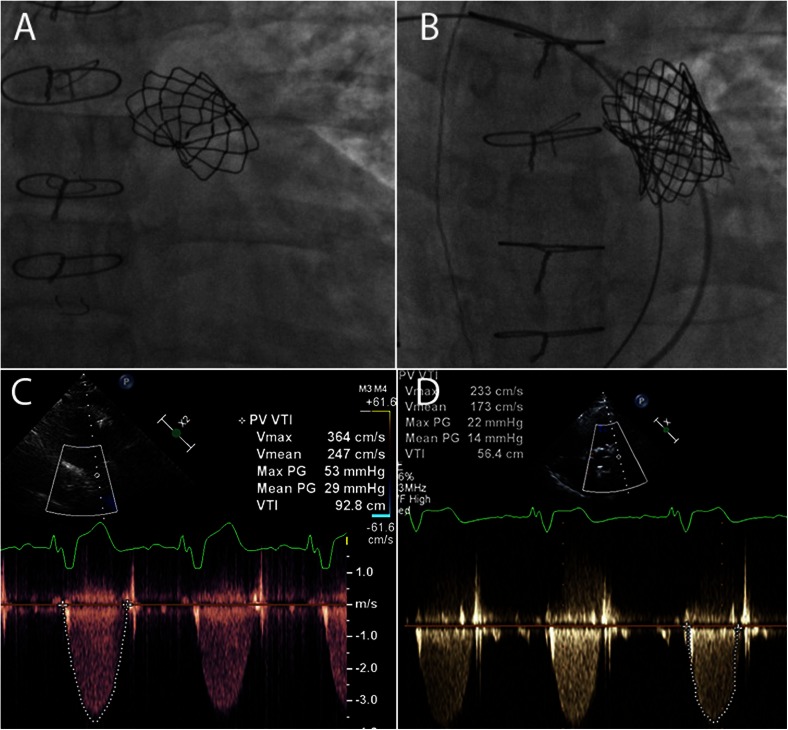
**a** Antero-posterior (AP) view of a fractured Melody transcatheter pulmonary valve (TPV) within a right ventricular to pulmonary artery homograft. **b** AP view of a deployed Melody TPV within the previously fractured Melody valve after pre-stenting. **c** Transthoracic echocardiographic image with continuous wave (CW) Doppler across the fractured Melody TPV demonstrating moderate stenosis (peak velocity 3.6 m/s, peak gradient of 53 mmHg, and mean gradient 29 mmHg). **d** Transthoracic echocardiographic image with CW Doppler signal across the deployed Melody valve within the previously fractured Melody valve showing significant improvement (reduction in velocity to 2.3 m/s, peak gradient to 22 mmHg and mean gradient to 14 mmHg)

**Fig. 6:**
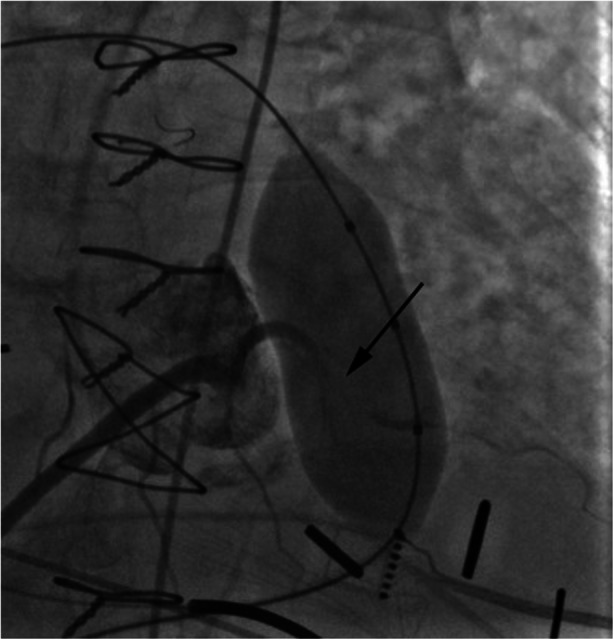
Antero-posterior (AP) angiographic view of aortic root injection with simultaneous balloon inflation across the RVOT showing coronary artery compression (*black arrow*) of an anomalous left anterior descending artery

**Fig. 7:**
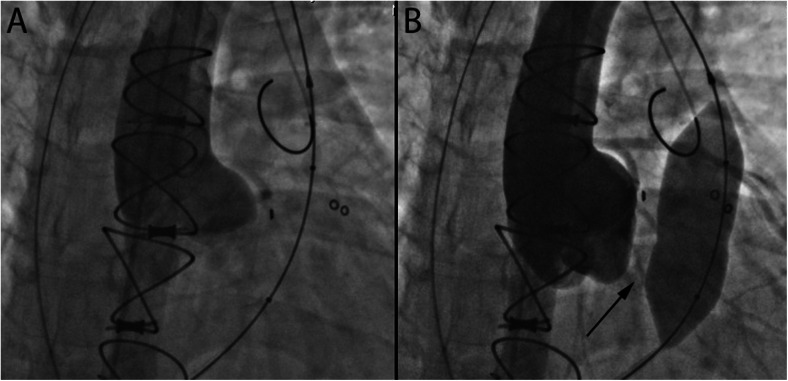
**a** Antero-posterior (AP) angiographic view of aortic root injection prior to balloon inflation within a dysfunctional bioprosthetic pulmonary valve. **b** Aortic root injection with simultaneous balloon inflation across the pulmonary valve showing aortic root compression and the development of aortic regurgitation (*black arrow*)

Stent fracture and endocarditis have been the primary causes for re-intervention [47, 55•, 57, 58, 59•]. Stent fracture often occurs in patients with conduits or native RVOT dysfunction and is likely due to repetitive stress on the platinum iridium frame. Pre-stenting before Melody valve placement has significantly reduced the incidence of stent fracture. Also, valve-in-valve implantation is a successful treatment option for Melody valve stent fracture (Fig. 5) [47, 55•, 60–62]. As for endocarditis, adherence with SBE prophylaxis is imperative; however, data suggests there is an increased risk of infective endocarditis with implanted Melody valves [57, 58, 59•]. Other complications such as conduit or pulmonary artery dissection or rupture are life threatening but rare and can be treated with covered stent placement. There is a 5 % risk of coronary artery compression, but this risk can be reduced by performing simultaneous balloon inflation in the RVOT with coronary or aortic root angiography to test for coronary artery compression (Fig. 6) [63]. Aortic compression may also occur in this population (Fig. 7). Lastly, valve embolization is a rare risk; which can be averted by adequate pre-procedural planning and RVOT sizing. However, when it does occur and the valve cannot be safely deployed in a distal pulmonary artery branch or vena cava, surgical removal may be necessary.

Transcatheter pulmonary valve replacement has also been successfully performed within failed bioprosthetic valves, not as part of RV-PA conduits, with excellent outcomes including freedom from repeat intervention of 92 % at 1 year and 92 % at 2 years [64]. The Melody valve can be utilized in most large diameter bioprostheses, up to a 24-mm maximal diameter, and the 26- or 29-mm Sapien valves can be used as well if the diameter is too large for a Melody valve [65].

**Fig. 8:**
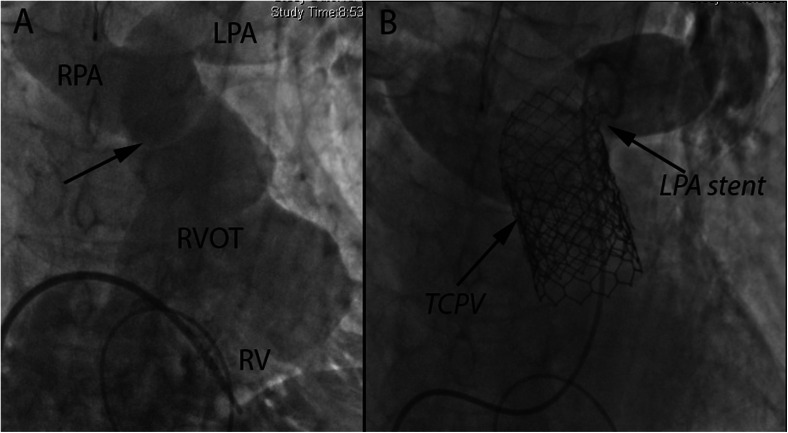
**a** Antero-posterior (AP) and cranial (CRA) angiographic view of a patient with transannular patch repair of the right ventricular outflow tract (RVOT) and main pulmonary artery (MPA) with a relative narrowing (*black arrow*) proximal to the bifurcation. The right ventricle (RV) is severely dilated. Right pulmonary arteries = RPA; left pulmonary artery = LPA. **b** Following pre-stenting with two stents, one extending into the LPA and “jailing” the RPA, but with open stent struts allowing antegrade bloodflow into the RPA. The Sapien transcatheter pulmonary valve (TPV) placement was deployed within the two stents. This technique allows for anchoring of the TPV in dilated RVOTs. Contrast injection in the LPA demonstrates a competent TPV without significant pulmonary regurgitation

In the subset of congenital heart disease patients with native RVOT or RVOT patch repairs, there is a growing body of literature on the utility of commercially available valves used off-label. In a retrospective trial of 31 patients, Melody pulmonary valve implantation in native RVOT due to either stenosis or regurgitation was safe and feasible; however, there was a high stent fracture rate of 32 % despite pre-stenting [66]. Boudjemline et al [67] showed that for patients with large RVOT, multiple stenting in Russian-doll style or jailing the PA method can create a suitable landing zone for the transcatheter valve (Fig. 8). Off-label use of the larger-diameter 29-mm Sapien XT and S3 valves in large native RVOT is being performed in select patients, and newer valves are being developed specifically for that indication [68].

The timing of pulmonary valve replacement in asymptomatic patients remains somewhat controversial [17, 25, 52, 53]. The advent of transcatheter valve therapies is in reality lowering the threshold for pulmonary valve replacement. Some experts argue that all patients with severe pulmonary regurgitation should undergo pulmonary valve replacement prior to the development of right ventricular dilation or dysfunction [17]. This aggressive early intervention approach must be weighed against the procedural risks, the potential for infective endocarditis, and the potential decrease in the internal orifice diameter of surgically placed conduits or bioprosthetic valves with multiple transcatheter pulmonary valve implants over a patient’s lifetime [16].

### Tricuspid Valve

**Fig. 9:**
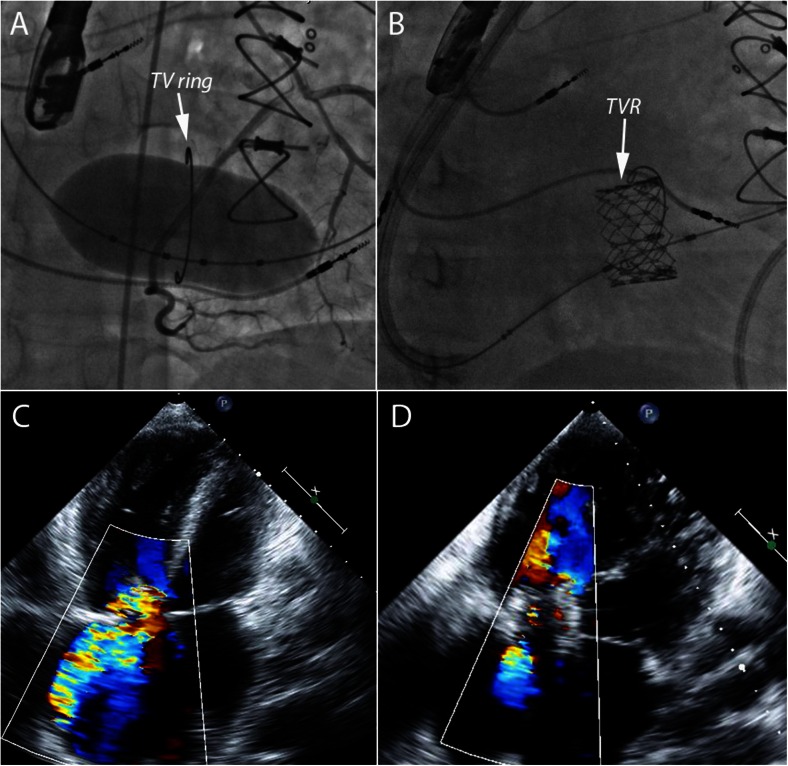
**a** Right anterior oblique (RAO) angiographic view of an inflated 30-mm balloon across a previously surgically placed incomplete tricuspid valve ring (*white arrow*). Simultaneous selective right coronary artery injection shows no evidence of compression. **b** Sapien transcatheter valve replacement (TVR) within the ring. **c** Transthoracic echocardiographic image (apical four-chamber view) with color Doppler flow showing severe tricuspid regurgitation across the native tricuspid valve. **d** Transthoracic echocardiographic image (apical four-chamber view) with color Doppler flow showing mild regurgitation across the Sapien valve

**Fig. 10:**
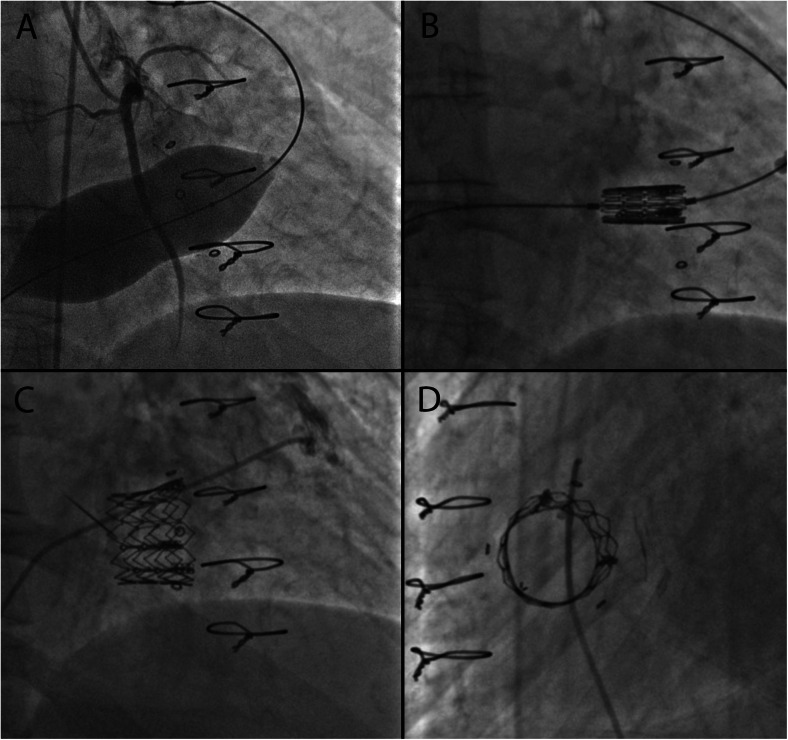
**a** Right anterior oblique (RAO) angiographic view of an inflated 30-mm Nucleus balloon (NuMED) across the surgically placed dysfunctional bioprosthetic Mosaic valve (Medtronic) in the tricuspid position. Simultaneous selective right coronary artery injection shows no evidence of compression. **b** Sapien valve being positioned across the bioprosthetic Mosaic valve. **c** RAO angiographic view of the deployed Sapien transcatheter valve in the tricuspid position. **d** Lateral angiographic view of the deployed Sapien valve in the tricuspid position

**Fig. 11:**
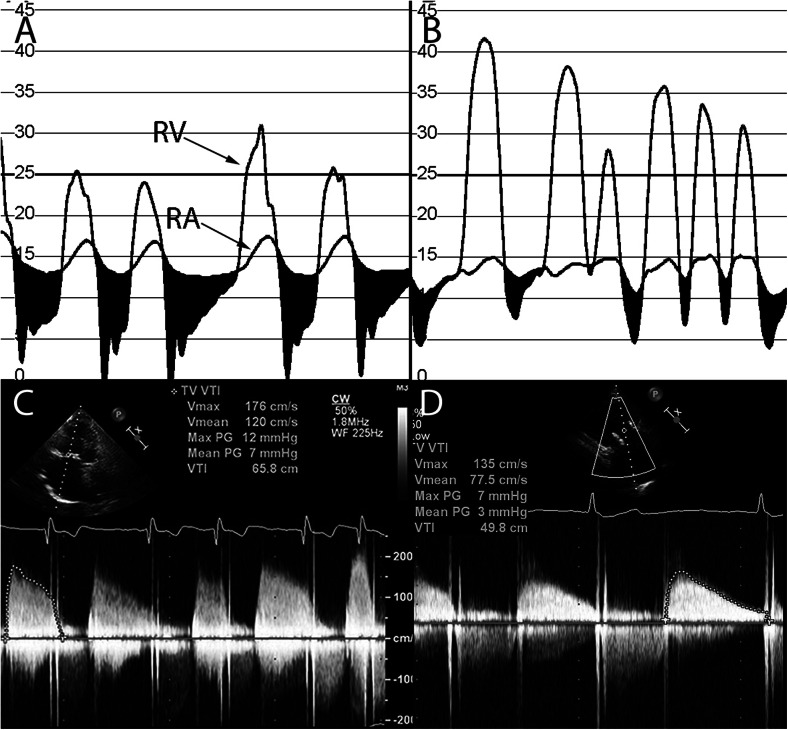
**a** Invasive hemodynamic tracings of simultaneous right ventricular and right atrial pressures in a patient with atrial fibrillation and a stenotic bioprosthetic tricuspid valve showing elevated diastolic gradients (*shaded black area*)—mean gradient 8 mmHg. **b** Invasive hemodynamic tracings of right ventricular and right atrial pressures showing reduction of tricuspid stenosis to the mild range (mean gradient 3 mmHg) after transcutaneous valve placement. **c** Transthoracic echocardiographic image with CW Doppler across the tricuspid valve showing moderate stenosis (mean gradient 7 mmHg). **d** Transthoracic echocardiographic image with CW Doppler across the tricuspid valve demonstrating reduction of mean gradient to 3 mmHg after transcutaneous valve placement

In congenital heart disease patients, prior surgical repair often involves annuloplasty rings with tricuspid valve repair, and less often tricuspid valve replacement. The risk of recurrent regurgitation in tricuspid valve repair is high and many will eventually require valve replacement [69]. Bioprosthetic TVR is associated with valvular stenosis and/or regurgitation as a long-term consequence [26, 27]. Transcatheter tricuspid valve replacement is feasible, and there is a growing body of evidence for the use of both the Melody and Sapien valves in failing tricuspid valve rings or bioprostheses (Figs. 9 and 10). The procedural success rate is high and immediate hemodynamic benefits are evident (Fig. 11); however, there is paucity of intermediate and long-term data [70–84]. The large-diameter Sapien valves have been successfully utilized in tricuspid annuloplasty rings and bands; however, the experience is limited to isolated case reports [80].

In native valves, the MitraClip (Evalve) has been used from a transjugular approach; however, the tricuspid valve morphology (three leaflets and chordal attachments to the right ventricular wall) makes it more difficult to successfully position the Mitraclip [60]. Other devices used include the Mitralign (Mitralign), a device originally designed to remodel the mitral annulus using pledgeted sutures, which has been successfully placed in some patients with severe tricuspid regurgitation [85]. The TriCinch (4Tech) [86], a percutaneous device designed for annular cinching of the tricuspid annulus, and the Millipede (Millipede), which involves the transcutaneous placement of a tricuspid annular ring with an attachment system, are still in pre-clinical trials.

Caval transcatheter valve implantation is another potential treatment option for severe tricuspid valve regurgitation, where valves are implanted in the superior and inferior vena cavae, with several case reports demonstrating hemodynamic success [83, 87, 88]. Clinical trials are currently investigating Sapien XT implantation in the inferior vena cava in patients with severe tricuspid regurgitation at prohibitive surgical risk (HOVER Clinical Trial NCT02339974, and TRICAVAL Clinical Trial NCT02387697).

## Conclusions

Right-sided transcatheter valvular implantation in patients with congenital heart disease and conduit or bioprosthetic valve dysfunction is not only gaining momentum with enormous potential for growth and innovation but is also quickly becoming the new standard of care. The minimally invasive and effective techniques of transcatheter valvular implantation have allowed for a reduction in the number of open cardiac surgeries that a patient with CHD can anticipate undergoing over a lifetime, therefore affecting the morbidity of this growing patient population. The use of TCPV in native RVOT is increasing, especially with the advent of large-diameter TCPV. There is a growing body of data on the efficacy and safety of transcatheter valve interventions in patients with tricuspid valve dysfunction. Pre-procedural planning with the use of multiple imaging modalities is integral to procedural success, and it is critical to combine both structural and functional data for determining the correct timing of any intervention. An interdisciplinary team approach is most ideal, with incorporation of hybrid procedures using both surgical and catheter-based techniques. A larger body of evidence with intermediate and long-term outcomes is needed to assess the longevity of these new techniques in comparison to the current surgical gold standard.

## References

[1] Elahi M, Dhannapuneni R, Firmin R (2005). Direct complications of repeat median sternotomy in adults. Asian Cardiovasc Thorac Ann.

[2] Temeck BK, Katz NM, Wallace RB (1990). An approach to reoperative median sternotomy. J Card Surg.

[3] Daebritz SH (2007). Update in adult congenital cardiac surgery. Pediatr Cardiol.

[4] van der Bom T, Zomer AC, Zwinderman AH (2011). The changing epidemiology of congenital heart disease. Nat Rev Cardiol.

[5] Verheugt CL, Uiterwaal CS, Grobbee DE (2008). Long-term prognosis of congenital heart defects: a systematic review. Int J Cardiol.

[6] Vida VL, Berggren H, Brawn WJ (2007). Risk of surgery for congenital heart disease in the adult: a multicentered European study. Ann Thorac Surg.

[7] Khairy P, Aboulhosn J, Gurvitz MZ (2010). Arrhythmia burden in adults with surgically repaired tetralogy of Fallot: a multi-institutional study. Circulation.

[8] Bonhoeffer P, Boudjemline Y, Saliba Z (2000). Percutaneous replacement of pulmonary valve in a right-ventricle to pulmonary-artery prosthetic conduit with valve dysfunction. Lancet.

[9] Coats L, Tsang V, Khambadkone S (2005). The potential impact of percutaneous pulmonary valve stent implantation on right ventricular outflow tract re-intervention. Eur J Cardiothorac Surg.

[10] Ong K, Boone R, Gao M (2013). Right ventricle to pulmonary artery conduit reoperations in patients with tetralogy of fallot or pulmonary atresia associated with ventricular septal defect. Am J Cardiol.

[11] Batlivala SP, Emani S, Mayer JE (2012). Pulmonary valve replacement function in adolescents: a comparison of bioprosthetic valves and homograft conduits. Ann Thorac Surg.

[12] Bouzas B, Kilner PJ, Gatzoulis MA (2005). Pulmonary regurgitation: not a benign lesion. Eur Heart J.

[13] Aboulhosn JA, Lluri G, Gurvitz MZ (2013). Left and right ventricular diastolic function in adults with surgically repaired tetralogy of Fallot: a multi-institutional study. Can J Cardiol.

[14] Fernandes FP, Manlhiot C, Roche SL (2012). Impaired left ventricular myocardial mechanics and their relation to pulmonary regurgitation, right ventricular enlargement and exercise capacity in asymptomatic children after repair of tetralogy of Fallot. J Am Soc Echocardiogr.

[15] Gatzoulis MA, Balaji S, Webber SA (2000). Risk factors for arrhythmia and sudden cardiac death late after repair of tetralogy of Fallot: a multicentre study. Lancet.

[16] Aboulhosn J, Levi DS (2015). Percutaneous pulmonary valve implantation: is earlier valve implantation better?. Circ Cardiovasc Interv.

[17] Borik S, Crean A, Horlick E (2015). Percutaneous pulmonary valve implantation: 5 years of follow-up: does age influence outcomes?. Circ Cardiovasc Interv.

[18] Ferraz Cavalcanti PE, Sa MP, Santos CA (2013). Pulmonary valve replacement after operative repair of tetralogy of Fallot: meta-analysis and meta-regression of 3,118 patients from 48 studies. J Am Coll Cardiol.

[19] Frigiola A, Tsang V, Bull C (2008). Biventricular response after pulmonary valve replacement for right ventricular outflow tract dysfunction: is age a predictor of outcome?. Circulation.

[20] Knauth AL, Gauvreau K, Powell AJ (2008). Ventricular size and function assessed by cardiac MRI predict major adverse clinical outcomes late after tetralogy of Fallot repair. Heart.

[21] Lee C, Kim YM, Lee CH (2012). Outcomes of pulmonary valve replacement in 170 patients with chronic pulmonary regurgitation after relief of right ventricular outflow tract obstruction: implications for optimal timing of pulmonary valve replacement. J Am Coll Cardiol.

[22] Oosterhof T, van Straten A, Vliegen HW (2007). Preoperative thresholds for pulmonary valve replacement in patients with corrected tetralogy of Fallot using cardiovascular magnetic resonance. Circulation.

[23] Therrien J, Siu SC, McLaughlin PR (2000). Pulmonary valve replacement in adults late after repair of tetralogy of fallot: are we operating too late?. J Am Coll Cardiol.

[24] Zdradzinski MJ, Qureshi AM, Stewart R (2014). Comparison of long-term postoperative sequelae in patients with tetralogy of Fallot versus isolated pulmonic stenosis. Am J Cardiol.

[25] Lurz P, Nordmeyer J, Giardini A (2011). Early versus late functional outcome after successful percutaneous pulmonary valve implantation: are the acute effects of altered right ventricular loading all we can expect?. J Am Coll Cardiol.

[26] Filsoufi F, Anyanwu AC, Salzberg SP (2005). Long-term outcomes of tricuspid valve replacement in the current era. Ann Thorac Surg.

[27] Garatti A, Nano G, Bruschi G (2012). Twenty-five year outcomes of tricuspid valve replacement comparing mechanical and biologic prostheses. Ann Thorac Surg.

[28] Zoghbi WA, Chambers JB, Dumesnil JG, et al. Recommendations for evaluation of prosthetic valves with echocardiography and doppler ultrasound: a report From the American Society of Echocardiography's Guidelines and Standards Committee and the Task Force on Prosthetic Valves, developed in conjunction with the American College of Cardiology Cardiovascular Imaging Committee, Cardiac Imaging Committee of the American Heart Association, the European Association of Echocardiography, a registered branch of the European Society of Cardiology, the Japanese Society of Echocardiography and the Canadian Society of Echocardiography, endorsed by the American College of Cardiology Foundation, American Heart Association, European Association of Echocardiography, a registered branch of the European Society of Cardiology, the Japanese Society of Echocardiography, and Canadian Society of Echocardiography. J Am Soc Echocardiogr. 2009;22(9):975–1014. 10.1016/j.echo.2009.07.013 19733789

[29] Zoghbi WA, Enriquez-Sarano M, Foster E (2003). Recommendations for evaluation of the severity of native valvular regurgitation with two-dimensional and Doppler echocardiography. J Am Soc Echocardiogr.

[30] Nishimura RA, Otto CM, Bonow RO (2014). 2014 AHA/ACC guideline for the management of patients with valvular heart disease: a report of the American College of Cardiology/American Heart Association Task Force on Practice Guidelines. J Thorac Cardiovasc Surg.

[31] Lancellotti P, Tribouilloy C, Hagendorff A (2010). European Association of Echocardiography recommendations for the assessment of valvular regurgitation. Part 1: aortic and pulmonary regurgitation (native valve disease). Eur J Echocardiogr.

[32] Vitarelli A, Mangieri E, Terzano C (2015). Three-dimensional echocardiography and 2D-3D speckle-tracking imaging in chronic pulmonary hypertension: diagnostic accuracy in detecting hemodynamic signs of right ventricular (RV) failure. J Am Heart Assoc.

[33] Habets J, Mali WP, Budde RP (2012). Multidetector CT angiography in evaluation of prosthetic heart valve dysfunction. Radiographics.

[34] Gopalan D (2011). Right heart on multidetector CT. Br J Radiol.

[35] Poterucha JT, Foley TA, Taggart NW (2014). Percutaneous pulmonary valve implantation in a native outflow tract: 3-dimensional DynaCT rotational angiographic reconstruction and 3-dimensional printed model. JACC Cardiovasc Interv.

[36] Cawley PJ, Maki JH, Otto CM (2009). Cardiovascular magnetic resonance imaging for valvular heart disease: technique and validation. Circulation.

[37] Morello A, Gelfand EV (2009). Cardiovascular magnetic resonance imaging for valvular heart disease. Curr Heart Fail Rep.

[38] Lewis MJ, O'Connor DS, Rozenshtien A (2014). Usefulness of magnetic resonance imaging to guide referral for pulmonary valve replacement in repaired tetralogy of Fallot. Am J Cardiol.

[39] Myerson SG (2009). Valvular and hemodynamic assessment with CMR. Heart Fail Clin.

[40] Warnes CA, Williams RG, Bashore TM (2008). ACC/AHA 2008 Guidelines for the Management of Adults with Congenital Heart Disease: Executive Summary: a report of the American College of Cardiology/American Heart Association Task Force on Practice Guidelines (writing committee to develop guidelines for the management of adults with congenital heart disease). Circulation.

[41] Hascoet S, Acar P, Boudjemline Y (2014). Transcatheter pulmonary valvulation: current indications and available devices. Arch Cardiovasc Dis.

[42] Zahn EM, Hellenbrand WE, Lock JE (2009). Implantation of the melody transcatheter pulmonary valve in patients with a dysfunctional right ventricular outflow tract conduit early results from the u.s. Clinical trial. J Am Coll Cardiol.

[43] Kenny D, Hijazi ZM, Kar S (2011). Percutaneous implantation of the Edwards SAPIEN transcatheter heart valve for conduit failure in the pulmonary position: early phase 1 results from an international multicenter clinical trial. J Am Coll Cardiol.

[44] Butera G, Milanesi O, Spadoni I (2013). Melody transcatheter pulmonary valve implantation. Results from the registry of the Italian Society of Pediatric Cardiology. Catheter Cardiovasc Interv.

[45] Faza N, Kenny D, Kavinsky C (2013). Single-center comparative outcomes of the Edwards SAPIEN and Medtronic Melody transcatheter heart valves in the pulmonary position. Catheter Cardiovasc Interv.

[46] McElhinney DB, Hellenbrand WE, Zahn EM (2010). Short- and medium-term outcomes after transcatheter pulmonary valve placement in the expanded multicenter US melody valve trial. Circulation.

[47] Armstrong AK, Balzer DT, Cabalka AK (2014). One-year follow-up of the Melody transcatheter pulmonary valve multicenter post-approval study. JACC Cardiovasc Interv.

[48] Eicken A, Ewert P, Hager A (2011). Percutaneous pulmonary valve implantation: two-centre experience with more than 100 patients. Eur Heart J.

[49] Lurz P, Coats L, Khambadkone S (2008). Percutaneous pulmonary valve implantation: impact of evolving technology and learning curve on clinical outcome. Circulation.

[50] Fraisse A, Aldebert P, Malekzadeh-Milani S (2014). Melody (R) transcatheter pulmonary valve implantation: results from a French registry. Arch Cardiovasc Dis.

[51] Vezmar M, Chaturvedi R, Lee KJ (2010). Percutaneous pulmonary valve implantation in the young 2-year follow-up. JACC Cardiovasc Interv.

[52] Batra AS, McElhinney DB, Wang W (2012). Cardiopulmonary exercise function among patients undergoing transcatheter pulmonary valve implantation in the US Melody valve investigational trial. Am Heart J.

[53] Coats L, Khambadkone S, Derrick G (2006). Physiological and clinical consequences of relief of right ventricular outflow tract obstruction late after repair of congenital heart defects. Circulation.

[54] Lurz P, Muthurangu V, Schuler PK (2012). Impact of reduction in right ventricular pressure and/or volume overload by percutaneous pulmonary valve implantation on biventricular response to exercise: an exercise stress real-time CMR study. Eur Heart J.

[55.•] McElhinney DB, Cheatham JP, Jones TK (2011). Stent fracture, valve dysfunction, and right ventricular outflow tract reintervention after transcatheter pulmonary valve implantation: patient-related and procedural risk factors in the US Melody Valve Trial. Circ Cardiovasc Interv.

[56] Cheatham JP, Hellenbrand WE, Zahn EM (2015). Clinical and hemodynamic outcomes up to 7 years after transcatheter pulmonary valve replacement in the US melody valve investigational device exemption trial. Circulation.

[57] Buber J, Bergersen L, Lock JE (2013). Bloodstream infections occurring in patients with percutaneously implanted bioprosthetic pulmonary valve: a single-center experience. Circ Cardiovasc Interv.

[58] McElhinney DB, Benson LN, Eicken A (2013). Infective endocarditis after transcatheter pulmonary valve replacement using the Melody valve: combined results of 3 prospective North American and European studies. Circ Cardiovasc Interv.

[59.•] Van Dijck I, Budts W, Cools B (2015). Infective endocarditis of a transcatheter pulmonary valve in comparison with surgical implants. Heart.

[60] Franzen O, von Samson P, Dodge-Khatami A (2011). Percutaneous edge-to-edge repair of tricuspid regurgitation in congenitally corrected transposition of the great arteries. Congenit Heart Dis.

[61] Nordmeyer J, Coats L, Lurz P (2008). Percutaneous pulmonary valve-in-valve implantation: a successful treatment concept for early device failure. Eur Heart J.

[62] Nordmeyer J, Lurz P, Khambadkone S (2011). Pre-stenting with a bare metal stent before percutaneous pulmonary valve implantation: acute and 1-year outcomes. Heart.

[63] Morray BH, McElhinney DB, Cheatham JP (2013). Risk of coronary artery compression among patients referred for transcatheter pulmonary valve implantation: a multicenter experience. Circ Cardiovasc Interv.

[64] Gillespie MJ, Rome JJ, Levi DS (2012). Melody valve implant within failed bioprosthetic valves in the pulmonary position: a multicenter experience. Circ Cardiovasc Interv.

[65] Finch W, Levi DS, Salem M (2015). Transcatheter melody valve placement in large diameter bioprostheses and conduits: What is the optimal "Landing zone"?. Catheter Cardiovasc Interv.

[66] Meadows JJ, Moore PM, Berman DP (2014). Use and performance of the Melody Transcatheter Pulmonary Valve in native and postsurgical, nonconduit right ventricular outflow tracts. Circ Cardiovasc Interv.

[67] Boudjemline Y, Brugada G, Van-Aerschot I (2012). Outcomes and safety of transcatheter pulmonary valve replacement in patients with large patched right ventricular outflow tracts. Arch Cardiovasc Dis.

[68] Cao QL, Kenny D, Zhou D (2014). Early clinical experience with a novel self-expanding percutaneous stent-valve in the native right ventricular outflow tract. Catheter Cardiovasc Interv.

[69] Celermajer DS, Bull C, Till JA (1994). Ebstein's anomaly: presentation and outcome from fetus to adult. J Am Coll Cardiol.

[70] Beckerman Z, Cohen O, Agmon Y (2013). Valve-in-valve in the tricuspid position for a stenosed bioprosthesis. Heart Surg Forum.

[71] Butcher CJ, Plymen CM, Walker F (2010). A novel and unique treatment of right ventricular inflow obstruction in a patient with a Bjork modification of the Fontan palliation before pregnancy. Cardiol Young.

[72] Calvert PA, Himbert D, Brochet E (2012). Transfemoral implantation of an Edwards SAPIEN valve in a tricuspid bioprosthesis without fluoroscopic landmarks. EuroIntervention.

[73] Condado J, Leonardi R, Babaliaros V (2015). Percutaneous tricuspid valve-In-ring replacement for the treatment of recurrent severe tricuspid regurgitation. Catheter Cardiovasc Interv.

[74] Cullen MW, Cabalka AK, Alli OO (2013). Transvenous, antegrade Melody valve-in-valve implantation for bioprosthetic mitral and tricuspid valve dysfunction: a case series in children and adults. JACC Cardiovasc Interv.

[75] Eicken A, Fratz S, Hager A (2010). Transcutaneous Melody valve implantation in "tricuspid position" after a Fontan Bjork (RA-RV homograft) operation results in biventricular circulation. Int J Cardiol.

[76] Godart F, Baruteau AE, Petit J (2014). Transcatheter tricuspid valve implantation: a multicentre French study. Arch Cardiovasc Dis.

[77] Hoendermis ES, Douglas YL, van den Heuvel AF (2012). Percutaneous Edwards SAPIEN valve implantation in the tricuspid position: case report and review of literature. EuroIntervention.

[78] Hon JK, Cheung A, Ye J (2010). Transatrial transcatheter tricuspid valve-in-valve implantation of balloon expandable bioprosthesis. Ann Thorac Surg.

[79] Mortazavi A, Reul RM, Cannizzaro L (2014). Transvenous transcatheter valve-in-valve implantation after bioprosthetic tricuspid valve failure. Tex Heart Inst J.

[80] Roberts PA, Boudjemline Y, Cheatham JP (2011). Percutaneous tricuspid valve replacement in congenital and acquired heart disease. J Am Coll Cardiol.

[81] Tzifa A, Momenah T, Al Sahari A (2014). Transcatheter valve-in-valve implantation in the tricuspid position. EuroIntervention.

[82] Tanous D, Nadeem SN, Mason X (2010). Creation of a functional tricuspid valve: novel use of percutaneously implanted valve in right atrial to right ventricular conduit in a patient with tricuspid atresia. Int J Cardiol.

[83] Laule M, Stangl V, Sanad W (2013). Percutaneous transfemoral management of severe secondary tricuspid regurgitation with Edwards Sapien XT bioprosthesis: first-in-man experience. J Am Coll Cardiol.

[84] Bentham J, Qureshi S, Eicken A (2013). Early percutaneous valve failure within bioprosthetic tricuspid tissue valve replacements. Catheter Cardiovasc Interv.

[85] Schofer J, Bijuklic K, Tiburtius C (2015). First-in-human transcatheter tricuspid valve repair in a patient with severely regurgitant tricuspid valve. J Am Coll Cardiol.

[86] Lauten A, Figulla HR (2015). Interventions at the tricuspid valve : What is possible?. Herz.

[87] Lauten A, Doenst T, Hamadanchi A (2014). Percutaneous bicaval valve implantation for transcatheter treatment of tricuspid regurgitation: clinical observations and 12-month follow-up. Circ Cardiovasc Interv.

[88] Lauten A, Ferrari M, Hekmat K (2011). Heterotopic transcatheter tricuspid valve implantation: first-in-man application of a novel approach to tricuspid regurgitation. Eur Heart J.

